# Polishing the Therapy of Onychomycosis Induced by *Candida* spp.: Amphotericin B–Loaded Nail Lacquer

**DOI:** 10.3390/pharmaceutics13060784

**Published:** 2021-05-24

**Authors:** Aleph M. S. Souza, Renato C. A. Ribeiro, Gleyse K. L. O. Pinheiro, Francisco I. Pinheiro, Wógenes N. Oliveira, Luanda B. F. C. Souza, André L. Silva, Lucas Amaral-Machado, Éverton N. Alencar, Guilherme M. Chaves, Eryvaldo S. T. Egito

**Affiliations:** 1 Graduate Program in Pharmaceutical Sciences, Federal University of Rio Grande do Norte (UFRN), Natal 59012-570, Brazil; alephmatthews03@gmail.com (A.M.S.S.); luanda_canario@hotmail.com (L.B.F.C.S.); guilherme.chaves@ufrnet.br (G.M.C.); 2 Graduate Program in Health Sciences, Federal University of Rio Grande do Norte (UFRN), Natal 59012-570, Brazil; recar_89@hotmail.com (R.C.A.R.); wogenes95@gmail.com (W.N.O.); machado.lucasam@gmail.com (L.A.-M.); 3 Graduate Program in Biotechnology, School of Health, Potiguar University (UnP)–Laureate International Universities, Natal 59056-000, Brazil; gleysekarina@msn.com (G.K.L.O.P.); irochima@gmail.com (F.I.P.); 4 Center for Biological Sciences and Health, Federal University of Western Bahia (UFOB), Barreiras 47800-000, Brazil; andre.leo17@gmail.com; 5 Graduate Program in Pharmaceutical Nanotechnology, Federal University of Rio Grande do Norte (UFRN), Natal 59012-570, Brazil; everton_alencar@hotmail.com

**Keywords:** amphotericin B, nail lacquer, extemporaneous product, onychomycosis, *Candida* spp., nail infection

## Abstract

Onychomycosis induced by *Candida* spp. has several limitations regarding its treatment. Nail lacquers display the potential to overcome these drawbacks by providing therapeutic compliance and increasing local drug bioavailability. Thus, this work aimed to produce a nail lacquer loaded with Amphotericin B (AmB) and evaluate its performance. The AmB-loaded nail lacquer was produced and preliminarily characterized. An AmB quantification method was developed. Stability, drug release, permeability and anti-*Candida* activity assays were conducted. The analytical method validation met the acceptance criteria. The drug loading efficiency was 100% (0.02 mg/g of total product), whereas the AmB stability was limited to ≅7 days (≅90% remaining). The nail lacquer displayed a drying time of 187 s, non-volatile content of around 20%*_w/w_*, water-resistance of approximately 2%*_w/w_* of weight loss and satisfactory in vitro adhesion. Moreover, the in vitro antifungal activity against different *Candida* spp. strains was confirmed. The AmB release and the ex vivo permeability studies revealed that AmB leaves the lacquer and permeates the nail matrix in 47.76 ± 0.07% over 24 h. In conclusion, AmB-loaded nail lacquer shows itself as a promising extemporaneous dosage form with remarkable anti-*Candida* activity related to onychomycosis.

## 1. Introduction

Onychomycosis is the general nomenclature used to describe any fungal nail infection [1] caused by different etiological agents. This infection affects 10% of the general population, and it is distributed worldwide [2]. In addition, onychomycosis can be diagnosed in all population groups, especially the elderly, on which its prevalence increases according to the age group: 20% in people older than 60 years and 50% in people older than 70 years [3,4]. This type of infection is also prevalent in individuals considered susceptible and at risk, such as immunosuppressed patients [5]. Although this infection is uncommon in children, the prevalence in this group is reaching 1.1% in North America, with similar findings observed in other countries [6].

This infection could affect both fingernail and toenail [3,4]. It could manifest itself as a distolateral subungual, superficial, endonyx, proximal subungual, mixed, totally dystrophic and secondary onychomycosis [1]. The prevalence in toenails is higher than in fingernails, which may be related to their slow growth rate, reduced blood supply and the dark and moist environment on shoes [3]. In addition, distolateral subungual onychomycosis, the most common subtype, represents a great challenge in treatment once the drug needs to permeate the nail plate when topical administration is used for its activity to occur [3]. Furthermore, other factors could be associated with the onychomycosis infections: (i) distorted nails, (ii) the use of closed shoes for long periods, (iii) nail trauma history, (iv) genetic predisposition, (v) hyperhidrosis, (vi) concurrent fungal infections and (vii) psoriasis [3,4,7].

Moreover, this infection causes damage in short or prolonged periods, leading to physical and psychological discomfort. As physical discomfort increases, the severity of outcomes progresses, mainly nail deformation, pain, disability, amputation or even death [8,9,10]. Psychological discomfort also affects patients, as they may experience embarrassment in social and work situations, particularly in those jobs in which public interaction with nails exposition is required [8,9,10]. 

Regarding the etiological agents commonly find in onychomycosis cases, three groups stand out: dermatophyte fungi, non-dermatophyte filamentous fungi or different kinds of yeasts [1]. The main dermatophyte fungi regarding this disease are fungus of the genders *Trichophyton* sp., *Epidermophyton* sp. and *Microsporum* sp. In addition, *Aspergillus* sp. and *Fusarium* sp. are the main genders representative of non-dermatophyte filamentous fungi, while *Candida* spp., *Malassezia* sp. and *Trichosporon* sp. are the main genders representative of the yeast group. Among which, *Candida* spp. have remarkable relevance. 

Although yeasts are usually part of the normal microbiota, if there is an imbalance added to predisposing factors and comorbidities, they can cause lesions at nails or surrounding tissues, thus, predisposing to onychomycosis. In this context, the role of the yeasts in onychomycosis cases corresponds up to 21.1% of the total cases. However, higher prevalence is becoming alarming in some countries [11]. In addition, elderly (6.07%) and at-risk individuals (mainly diabetic (3.97%) and psoriatic patients (3.35%)), immunosuppressed patients (1.72%) and dialysis patients (0.92%) have the highest prevalence of onychomycosis induced by yeasts [12]. As previously mentioned, *Candida* spp. has remarkable prominence in onychomycosis cases and scientific reports. This suggests that the prevalence of this gender can reach more than 50% of the onychomycosis cases induced by yeasts [13]. In this context, *C. albicans* and *C. parapsilosis*, followed by *C. tropicalis*, are the most prevalent species [2,13,14,15,16,17,18,19].

In general, oral antifungals, such as terbinafine and itraconazole, are the first choice of treatment [20,21]. Nonetheless, they present several side effects, drug interactions (drug–drug or drug–food) and long treatment time, impairing patient compliance and leading to high rates of disease recurrence. In addition, these prolonged treatments are associated to the development of new diseases, such as chronic or acute liver disease and renal failure. [10,20,21,22,23]. 

**Scheme 1:**
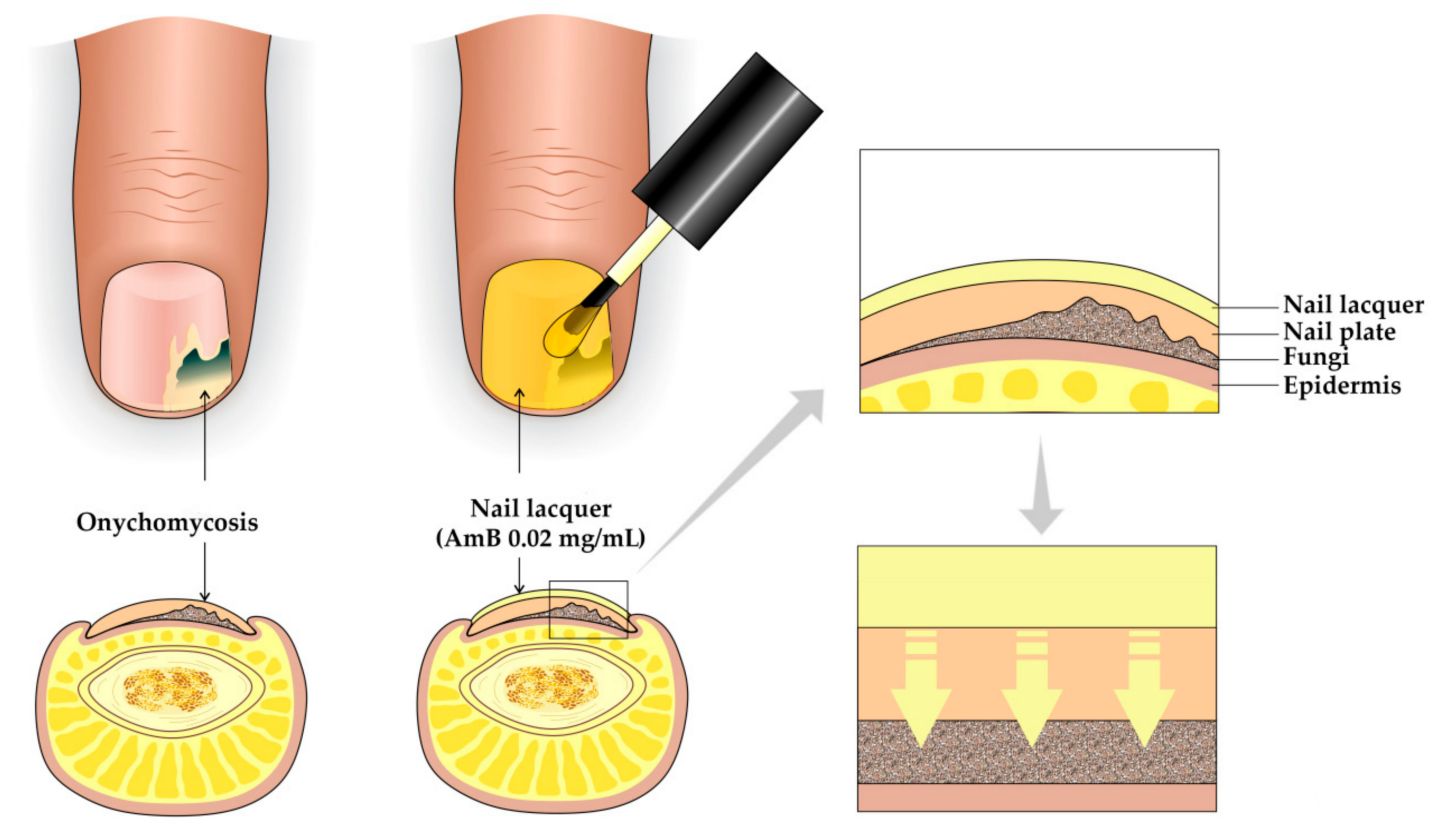
Overall ungueal anatomy and its correlation to the mechanism of action of nail lacquers for the treatment of onychomycosis.

The topical treatment uses amorolfine, ciclopirox, efinaconazole or tavaborole as drugs of choice [21,24]. However, some of these drugs are available in pharmaceutical dosage forms unsuitable for nails, e.g., creams, lotions, gels, solutions or suspensions [21,24]. In addition, to ensure nail porosity and, consequently, better drug penetration, these topical formulations also require abrasion processes in the nail matrix before application [21,24,25]. Hence, lacquers have been formulated to provide better penetration of active substances through the nail plate, while lacquer inactive substances (vehicles) maintain the proper concentration of the active substances on the nail surface (Scheme 1), which increases the effectiveness of the antifungal agent [25].

However, since most antifungal drugs present poor permeability in the nail site, which can be associated with the drugs’ molecular weight, with their ionic charge and also with the vehicle ionic strength, their topical use requires constant reapplication to promote the pharmacological effect, which may also result in low therapeutic compliance [26,27]. In addition to the drawbacks of topical formulations, nail abrasion before administration is also required, making the treatment even more complex [24].

Based on these remarks, it is possible to suggest that the use of an antifungal agent by topical route, delivered by a suitable formulation with enhanced permeation and higher fungicidal activity than the aforementioned molecules could overcome most of the described drawbacks. Accordingly, amphotericin B (AmB) can be highlighted among the currently approved molecules. AmB is an antifungal (and antiparasitic) drug that presents a critical fungicidal effect with broad spectrum of action and high therapeutic effectiveness [26,28]. Nevertheless, the high molecular weight of this molecule can difficult the permeation across the nail plate. Compared to other antifungals, AmB is used as an immediate-acting fungicide, since its primary mechanism of action involves the direct drug interaction with the fungal cell wall via ergosterol, the main sterol compound presented in the cell membrane of fungi. This interaction promotes pores formation, which leads to the influx of water, ions and low-molecular-weight polar proteins. Hence, the pathogen survival is compromised [29,30]. In addition, this drug presents remarkable antifungal effectiveness in onychomycosis clinical practice, as already reported by the literature findings [31]. 

Considering the above-mentioned AmB advantages and disadvantages, this work was developed based on the hypothesis that the AmB loading in a nail-lacquer formulation could improve the topical treatment of onychomycosis, in vitro, in which a single dose of the lacquer would lead to the microorganisms’ death without the need for multiple applications. The rationale behind this approach is that the AmB incorporated into the nail lacquer would be free to permeate the nail matrix and to reach the pathogens due to the lacquers’ physicochemical characteristics. Therefore, the purpose of this work was to load the AmB into a marketed nail-lacquer base formulation to produce an AmB-loaded nail lacquer as a new AmB dosage form for the management of onychomycosis induced by *Candida* spp., to perform its physicochemical characterization and stability study, to assess the in vitro antifungal activity and to determine its drug release and permeability.

## 2. Materials and Methods

### 2.1. Materials 

Chemicals: Agar Müeller Hinton and Saboraud Dextrose Agar and Broth were purchased from HiMedia^®^ (Mumbai, India). Amphotericin B (AmB) was obtained from Infect Chemphar CO. (Hong Kong, China). Dimethyl sulfoxide (DMSO) 99.9% A.C.S spectrophotometric grade, Tween^®^ 80 and sodium phosphate dibasic were provided from Sigma-Aldrich (Saint Louis, MO, USA). The colorless nail lacquer was purchased from Ideal Cosméticos (Nova Lima, Brazil). Anhydrous monobasic potassium phosphate and glycerol came from Synth (São Paulo, Brazil). Sodium chloride and potassium chloride were provided from Vetec (Duque de Caxias, Brazil) and QEEL (São Paulo, Brazil), respectively.

Microorganisms: The four American Type Culture Collection strains of *Candida* spp. (*Candida albicans* ATCC 90028, *C. glabrata* ATCC 2001, *C. tropicalis* ATCC 13803 and *C. parapsilosis* ATCC 22019) and one Centraalbureau *voor Schimmelcultures (C. dubliniensis* CBS 7987) were donated by the culture collection of the Laboratory of Medical and Molecular Mycology from the Federal University of Rio Grande do Norte (UFRN, Natal, Brazil). The microorganisms were maintained in Saboraud Dextrose Broth containing 20% glycerol, frozen at −80 °C until the moment of the experiment. All stored fungi were cultured in Saboraud Dextrose Agar for 48 h at 37 °C before the experiments. 

### 2.2. Methods

#### 2.2.1. Amphotericin B–Loaded Nail Lacquer’s Production

The AmB-loaded nail lacquer was produced with a drug concentration of 0.02 mg/g of nail lacquer. First, a fresh AmB solution at 0.4 mg/mL was prepared in DMSO. Then, 250 μL of this solution was added to 5.0 g of colorless nail lacquer (Table 1), under moderate magnetic stirring (C-MAG HS 7 IKA^®^, Campinas, Brazil), for 15 min. 

#### 2.2.2. Validation of the Analytical Method

A UV–Vis spectrophotometry method was used for AmB quantification in the nail lacquer. First, a wavescan (λ = 300–450 nm) was performed to determine the maximum absorbance wavelength (λ_max_) from an AmB solution in DMSO (0.02 mg/mL). The solvent itself was used as blank (UV–Vis spectrophotometer Mod. Libra S32, Biochrom, Holliston, USA) [43]. The validation followed the specifications of the International Council for Harmonization (ICH) [43,44]. The colorless nail lacquer (without AmB) in DMSO (at the same ratio of the tested sample) was used as blank for the accuracy, robustness and precision experiments. AmB at 0.5 µg/mL diluted in DMSO from the sample target concentration (0.02 mg of AmB/g of nail lacquer) was used as the target analytical sample.

##### Specificity

A total of 250 μL of nail lacquer samples (with and without AmB) was diluted in 10 mL of DMSO. Both dilutions were placed into 10 cm quartz spectrophotometer cuvettes and a wavescan from λ = 300 to 450 nm was obtained. The colorless nail lacquer in DMSO (at the same ratio of the tested sample) and pure DMSO were used as blanks for this experiment. The obtained spectra were analyzed at 416 nm (λ_max_) to infer the method’s specificity and further parameters [43,44]. The experiment was conducted in triplicate.

##### Linearity and Range

The experiment started from a 0.4 mg/mL AmB in DMSO solution [45] and successive dilutions were made by using the stationary cuvette method [46]. The AmB concentration ranged from 0.12 to 1.11 µg/mL [43,44]. A calibration curve was plotted in the range that exhibited adequate linearity for further AmB quantification calculations.

##### Detection Limit (DL) and Quantification Limit (QL)

DL was calculated according to Equation (1), whereas QL was calculated according to Equation (2):

(1) $$\mathrm{DL}\;=3.3\;\frac{S}{M}\;\;$$

(2) $$\mathrm{QL}=10\;\frac{S}{M}\;$$

wherein *S* is the standard deviation of the calibration curves’ intercepts, and *M* is the Slope of the calibration curve.

##### Precision and Accuracy/Recovery

The target analytical sample was used across the experiments. Repeatability (intra-day) was performed by quantifying the test sample 9 times. Intermediate precision was performed by quantifying the test sample in two different ways: (i) in triplicate for three consecutive days (inter-day); or (ii) 9 times by three different analysts (inter-analyst). 

The accuracy was inferred by the percent nominal mean (% Recovery) of 3 determinations for the target analytical sample [43,44].

##### Robustness

Variations of solvent and temperature were used for the experiments: (i) two batches of DMSO from different manufacturers and (ii) two temperatures (15 ± 2 °C and 30 ± 2 °C) for the spectroscopic conditions. The analytical response according to the parameters variation was evaluated for the target analytical sample. 

#### 2.2.3. Amphotericin B–Loading Evaluation

The AmB content in the nail-lacquer samples was evaluated after a dilution of 250 μL of AmB-loaded nail lacquer in 10 mL of DMSO. The colorless nail lacquer (without AmB) in DMSO (at the same ratio of the tested sample) was used as blank. Furthermore, the AmB loading efficiency in the nail lacquer was determined by comparing the concentration of the total amount of AmB-loaded nail lacquer in the bulk and in the supernatant. The latter was obtained by submitting the product to centrifugation in an Eppendorf^®^ Centrifuge (AG, 5418 R, Germany) at 5 °C at 9.000 G for 10 min. Then, aliquots of 250 μL, before centrifugation (AmBBulk) and 250 μL of the supernatant after centrifugation (AmBSob) were analyzed at the same conditions of the calibration curve. All experiments were performed in triplicate. The percentage of loading capacity was calculated according to Equation (3) [45]:

(3) $$\mathrm{LC}\;\left(\%\right)=\frac{\mathrm{AmBSob}}{\mathrm{AmBBulk}}\;\times \;100$$

where in LC (%) means loading capacity, AmBSob means concentration of AmB in the supernatant and AmBBulk means concentration of AmB before centrifugation.

#### 2.2.4. Amphotericin B–Stability Evaluation

The AmB-loaded nail lacquer was produced and stored in hermetically sealed glass vials at 5 ± 3 °C. Stability parameters were chosen based on the recommended storage temperature for the pure AmB and following the specification of the International Council for Harmonization (ICH) in the Technical Requirements for Pharmaceuticals for Human Use guideline (*Stability Testing of New Drug Substances and Products Q1A(R2)*). The drug content of samples was analyzed (at 0, 7, 15, 30, 60 and 90 storage days) according to Section 2.2.2 and by the AmB spectrum recording from 300 to 450 nm [47]. The analysis was performed in triplicate to evaluate the drug content decay according to the chemical changes in this drug’s chromophore.

#### 2.2.5. Physicochemical Characterization of the AmB-Loaded Nail Lacquer

**Scheme 2:**
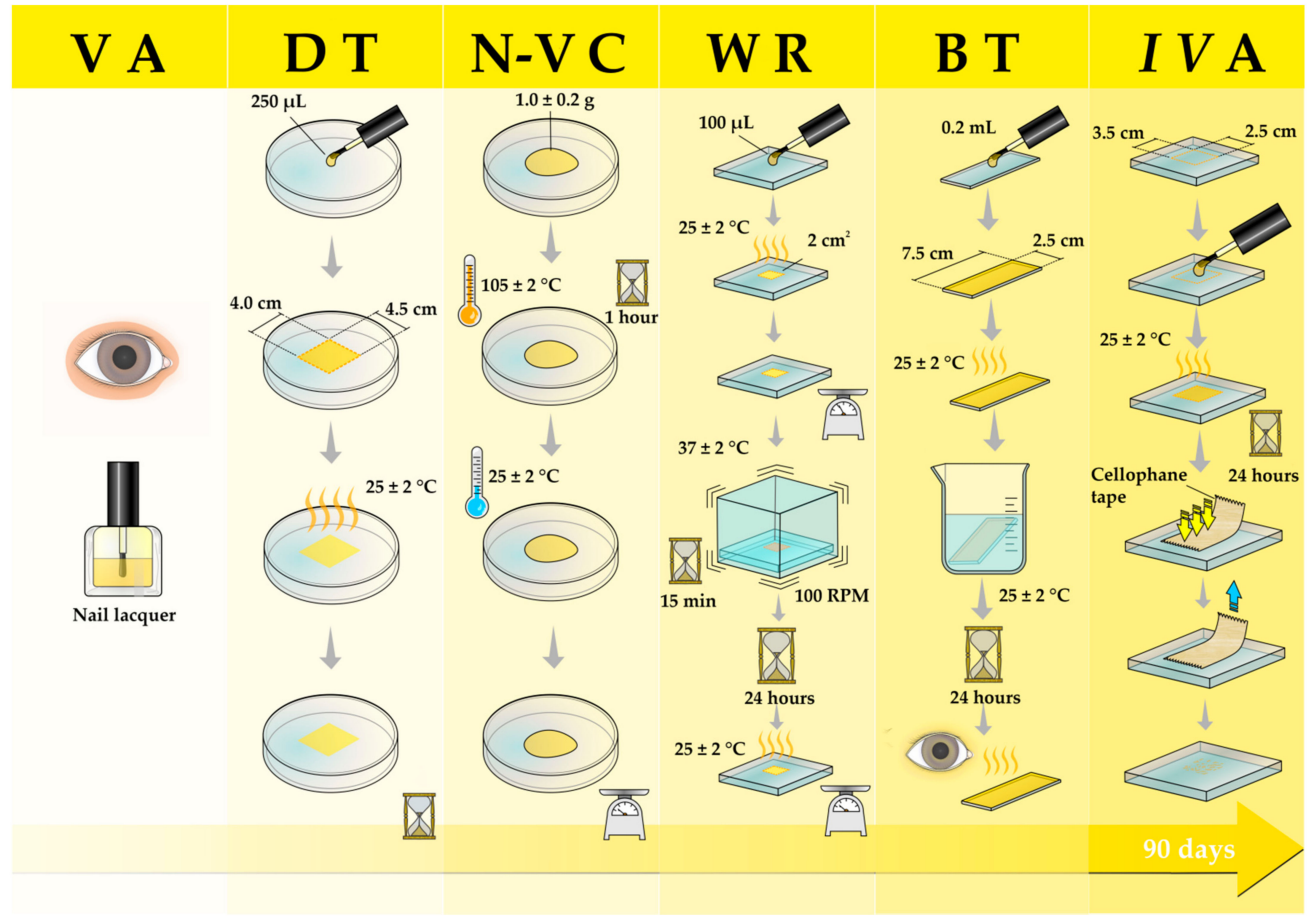
Physicochemical characterization of the AmB-loaded nail lacquer. VA, visual aspects; DT, drying-time test; N-VC, non-volatile content test; WR, water-resistance test; BT, blush test; and IVA, in vitro adhesion test.

In order to evaluate the physicochemical characteristics of the AmB-loaded nail lacquer at storage conditions. The following tests were performed over 90 days (Scheme 2).

##### Visual Aspects

The samples were analyzed by visual inspection to observe changes in color, visual precipitate formation and nail-lacquer unevenness.

##### Drying-Time Test

The method was carried out through the application and spreading of the AmB-loaded nail lacquer (250 μL) in a limited area (4.0 × 4.5 cm) of a petri dish, left to dry at 25 ± 2 °C [48]. Then, the drying time was measured by a stopwatch when obtaining a solid translucent film [48]. The analysis was performed in triplicate.

##### Non-Volatile Content Test

An amount of 1.0 ± 0.2 g of the AmB-loaded nail lacquer was weighed and spread in a petri dish [48]. Then, the plate was placed in an oven NI1514 (NOVA instruments, Piracicaba, Brazil) at 105 ± 2 °C for 1 h. Posteriorly, the plate was removed from the oven, cooled to 25 ± 2 °C and weighted. Non-volatile content was determined by the initial and final weight difference [48]. The analysis was performed in triplicate.

##### Water-Resistance Test

The test was carried out by spreading an aliquot of AmB-loaded nail lacquer (100 μL) over a glass surface area of 2 cm^2^ and drying at 25 ± 2 °C [48]. The sample-containing glass was weighed and then immersed in a thermostatic bath under magnetic stirring (C-MAG HS 7, IKA^®^, Wilmington Delaware USA) at 37 ± 2 °C for 15 min at 100 RPM. After 24 h, the glass was removed from the bath, left to dry at room temperature (25 ± 2 °C) and, then weighed. Water-resistance was determined by initial and final weight difference, and the results were expressed in weight loss (%) [48]. The analysis was performed in triplicate.

##### Blush Test

The blush test was performed to evaluate any blistering or peeling off in the nail lacquer after water contact [48]. Initially, 0.2 mL of AmB-loaded nail lacquer was uniformly spread on a 7.5 × 2.5 cm glass slide. Then, samples were dried at 25 ± 2 °C to obtain a solid film. The slide was submerged into tap water in a beaker at 25 ± 2 °C without stirring. After 24 h, the plate was removed from the water and left to dry at room temperature for 4 h. Posteriorly, the plates were evaluated by visual inspection and the occurrence of any blistering or peeling off in the nail lacquer after contact with water implied failing to meet the specifications [48]. The analysis was performed in triplicate.

##### In Vitro Adhesion Test

To evaluate the AmB-loaded nail lacquer’s adhesion ability [48,49], an area of 3.5 × 2.5 cm was delimited on a glass plate, and a standardized drop (100 µL) of the sample was applied and spread out. Samples were dried at 25 ± 2 °C for 24 h until a solid film was formed. Then, the film was covered with cellophane tape, in which a pressure was manually applied to ensure the contact, leaving a piece of free unadhered tape tab to be manually peeled off from the nail lacquer film, at an angle of approximately 60 ° [48,49].

The area containing the remaining film adhered to the glass plate was calculated by the square counting method and the percentage of the film removal was determined in scores, as follows: (0) nail-lacquer film unaffected; (1) some small parts of the film were detached at the intersections of the squares, with less than 5% of the total nail-lacquer film area affected; (2) some small parts of the film were detached along the edges and/or at the intersections of the squares with affected area ≥5% and ≤15%; (3) the nail-lacquer film was partially or entirely detached in large lines, and/or in different parts of the squares, with affected area ≥15% but ≤35%; (4) the nail-lacquer film was detached in large lines, and/or in different parts of the squares, with affected area ≥35% and ≤65%; (5) any degree of flaking or detachment that could not be classified under 4 [48,49]. Hence, the higher score is indicative of poor adhesion ability [48,49].

#### 2.2.6. In Vitro Antifungal Activity Evaluation

The method was carried out according to the Clinical and Laboratory Standards Institute (CLSI) guideline, *Method for Antifungal Disk Diffusion Susceptibility Testing of Yeasts (M44-A2)* with modifications [50]. Briefly, standardized fungal inocula (adjusted to 0.5 MacFarland) were seeded on the surface of petri dishes containing Müeller Hinton Agar. Nine 10 mm–diameter wells were cut on the agar, where freshly manufactured samples and controls (volume = 100 μL and AmB content = 2 μg) were placed. Plates were incubated at 35 ± 1 °C for 48 h. Growth inhibition halos were measured in millimeters, using a halometer [7,50].

The blank nail-lacquer formulation (N), the nail lacquer added of DMSO (at the same proportion used to load AmB-ND), AmB/DMSO solution at 0.02 mg/mL (AD), pure DMSO (D), Fungizone^®^ (AmB water dispersion) at 0.02 mg/mL (F) and saline solution at 0.9% *_w/v_* (S) were used as controls. The tested sample was AmB-loaded nail lacquer at 0.02 mg/mL (AND). The experiment was performed in triplicate.

#### 2.2.7. In Vitro AmB-Loaded Nail Lacquer’s Drug Release

**Scheme 3:**
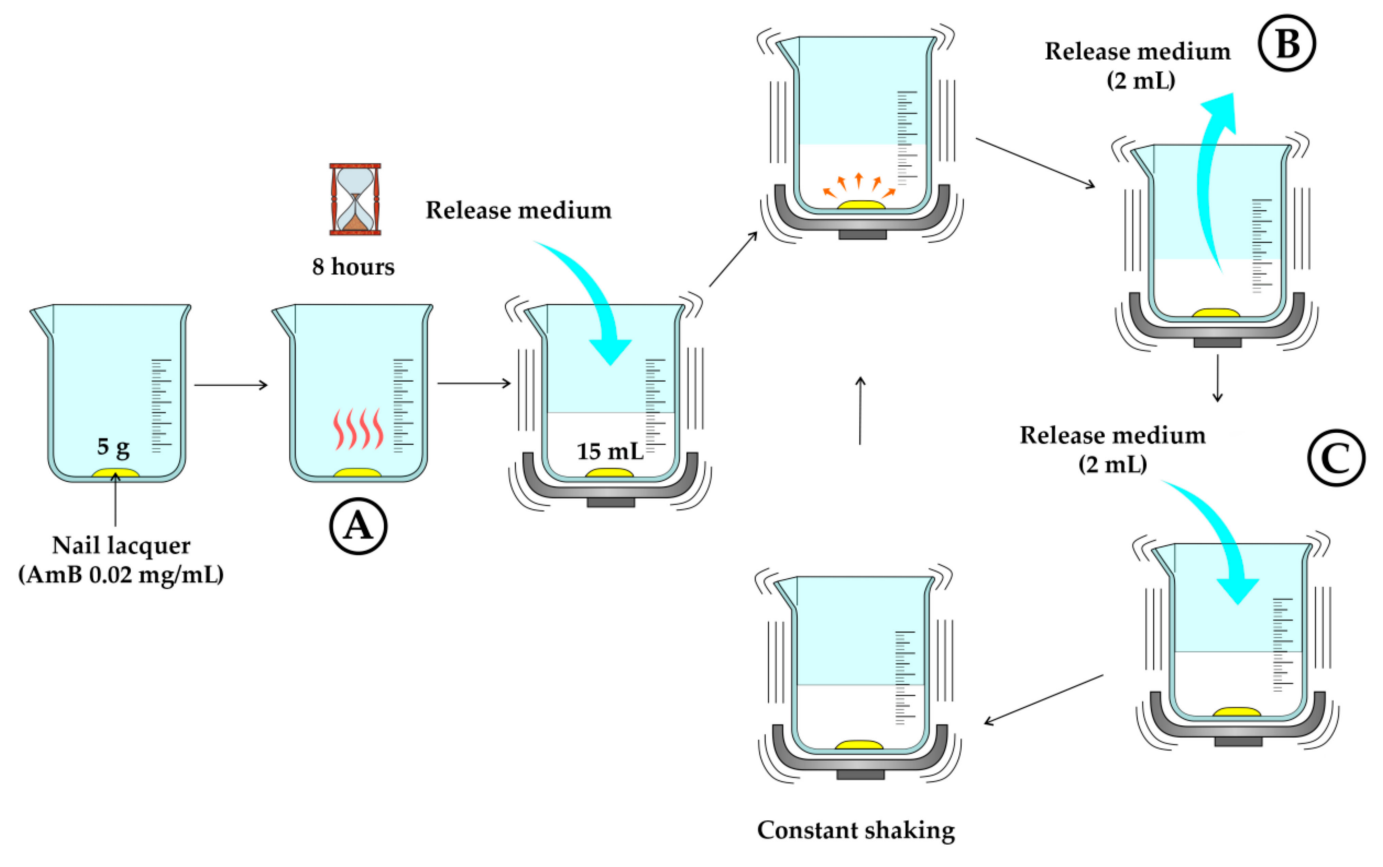
Graphical illustration of the in vitro AmB-loaded nail lacquer’s drug release. (**A**) The nail lacquer was applied and left to dry for 8 h. For the release assay, 2 mL of the medium was collected at each time interval for AmB quantification (**B**) and immediately replaced by fresh medium (**C**).

The in vitro AmB release was performed according to Kim et al. (2010) [51] with modifications (Scheme 3). The goal of this experiment, which was similar to a dissolution assay, was to evaluate the release of the drug from the nail-lacquer matrix and to investigate if the drug amount necessary to promote an antifungal effect was released from the product. The experiment was performed by using 5 g of the AmB-loaded nail-lacquer formulation (0.02 mg/g) added to a beaker and left to dry for 8 h. Posteriorly, 15 mL of the release medium (0.1 M phosphate saline buffer, pH 7.4 + 2% *_w/v_* of Tween^®^ 80) was carefully added and remained under constant shaking (100 RPM), using an incubator TE-420 (Tecnal, São Paulo, Brazil) at 37 ± 0.5 ° C, providing sink conditions for AmB release. For the AmB quantification, 2 mL of the release medium was collected and immediately replaced by a new and fresh medium. These experimental conditions were maintained for 24 h and the AmB quantification was performed at previously determined time intervals (0.00, 0.25, 0.50, 0.75, 1, 2, 3, 6, 9, 12 and 24 h).

Additionally, to measure the AmB released from the AmB-loaded nail lacquer, a control sample (representative of 100% of released AmB) was used (250 µL of AmB/DMSO solution at 0.4 mg/mL + 15 mL of the release medium). Henceforward, the samples were collected at the determined time intervals and had their absorbance measured by using UV–Vis. The AmB-loaded nail lacquer release was calculated as showed in Equation (4). The cumulative drug release was calculated as the sum of the previous drug release percent at each point. The samples collected from the third hour of experiment, in which the amount of AmB was too high, underwent dilution (250 µL) in fresh medium (10 mL) to assure compliance to the Beer–Lambert Law in UV–Vis readings. All experiments were performed in triplicate.

(4) $$\mathrm{Drug}\;\mathrm{release}\;\mathrm{at}\;\mathrm{time}\;X\;\left(\%\right)=\frac{\mathrm{Abs}.\mathrm{SampletimeB}\;-\mathrm{Abs}.\mathrm{SampletimeA}}{\mathrm{Abs}.\mathrm{Control}}\times 100$$

where Abs.Sample_timeB_ = absorbance in the predetermined time, Abs.Sample_timeA_ = absorbance in the time before and Abs.Control = absorbance from the control sample.

#### 2.2.8. Ex Vivo AmB-Loaded Nail Lacquer’s Drug Permeation

The permeability of the AmB-loaded nail lacquer was assessed by using bovine hooves as an ex vivo animal nail matrix model, and Franz vertical diffusion cells as a permeation apparatus. The hooves were purchased as cleaned and cartilage/connective tissue-free products. Their thickness was standardized at approximately 60 µm of diameter by a HistoCore MULTICUT semi-automated rotating microtome (Leica Biosystems, United States). Then, flat discs (2.5 mm of diameter) were cut and inspected for pores or any irregularities by visual inspection before use.

The receiver compartment (10 mL of volume) from the Franz diffusion cell was filled with 0.1 M phosphate saline buffer, pH 7.4 + 2% *_w/v_* of Tween^®^ 80. The medium remained under constant magnetic stirring at 100 RPM (digital magnetic stirrer RO 15, IKA, United States) and the temperature was set at 37 °C by a water circulation pump associated with a thermostatic bath. Thus, an aliquot of the AmB-loaded nail lacquer (4 g) was added to the donor compartment and 1 mL of medium was collected from the receiver compartment at previously determined time intervals (0.00, 0.25, 0.50, 0.75, 1, 2, 3, 6, 9, 12 and 24 h) for absorbance readings. The assay was conducted in triplicate and under sink conditions [48,52].

AmB in DMSO solution (at the same experimental concentration and conditions) was used for relative percent calculation as a control sample (representative of 100% of permeated AmB). The AmB-loaded nail lacquer’s permeation was calculated according to the Equation (5) and the cumulative drug permeated means the sum of the previous drug permeation percent at each point.

(5) $$\mathrm{Drug}\;\mathrm{permeation}\;\mathrm{at}\;\mathrm{time}\;X\;\left(\%\right)=\frac{\mathrm{Abs}.\mathrm{SampletimeB}\;-\mathrm{Abs}.\mathrm{SampletimeA}}{\mathrm{Abs}.\mathrm{Control}}\times 100$$

where Abs.Sample_timeB_ = absorbance in the predetermined time, Abs.Sample_timeA_ = absorbance in the time before and Abs.Control = absorbance from the control sample.

#### 2.2.9. Statistical Analysis

Statistical analysis was performed by using the software GraphPad Prism^®^ version 5.01. Student’s *t*-test (for pairs of groups) and one-way analysis of variance test (ANOVA, for multiple groups) were used to determine the level of statistical significance between the groups. The *p*-value < 0.05 was considered statistically significant.

## 3. Results

### 3.1. AmB-Loaded Nail Lacquer’s Quantification and Stability Evaluation

To provide a suitable AmB quantification from the AmB-nail lacquer, an analytical method validation was performed by determining specificity, linearity, range, accuracy, precision, robustness, detection and quantification limits [44] (Table 2).

The specificity evaluation revealed that the components presented on the nail-lacquer formulation did not interfere in the absorbance of AmB at 416 nm (*p*-value > 0.05). Moreover, the linearity was determined by using an AmB calibration curve with concentrations from 0.12 to 1.11 µg/mL (y = 0.9701x + 0.0262). The obtained determination coefficient (R^2^) was 0.9997. In addition, the detection and quantification limits were 0.073 µg/mL and 0.221 µg/mL, respectively. Furthermore, the accuracy was assessed based on the recovery percent (102.07 ± 1.23). Finally, the intra-day precision assay demonstrated the method’s high reliability, while the inter-day and inter-analyst assays demonstrated that the method can be executed by different analysts and conducted over different days with reproducibility. Overall, the method was considered validated and proper for AmB quantification.

**Figure 1:**
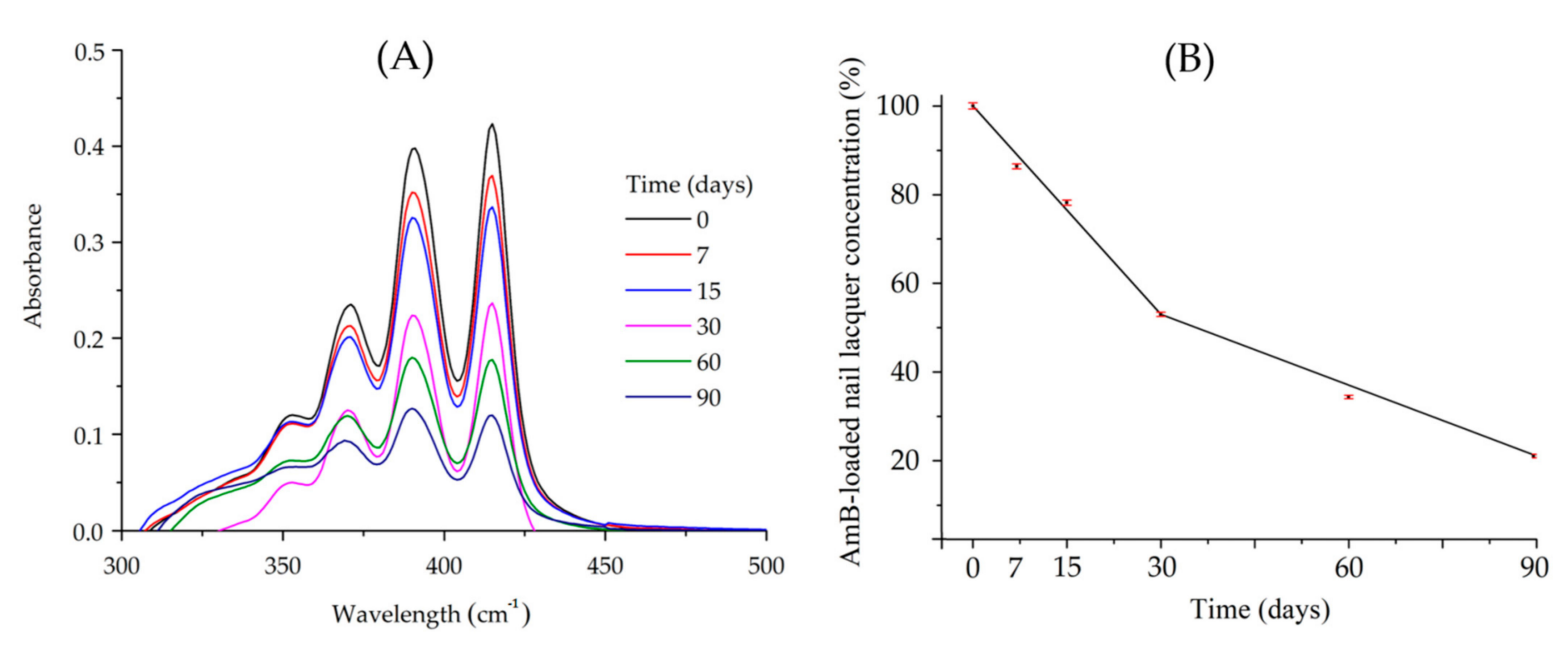
Spectroscopic signal emitted by the AmB’s chromophore group over 90 days. (**A**) Wavescan profile of AmB-loaded nail lacquer. (**B**) AmB-loaded nail lacquer’s concentration over time.

Furthermore, the AmB-loading capacity was evaluated (99.8 ± 0.5%) right after the AmB-nail-lacquer production. The formulations were also stored at 5 ± 3 °C and protected from light to be analyzed for 90 days regarding AmB content. The absorbance profile, recorded as a wavescan between 300 and 450 nm (Figure 1A), showed a decrease in the spectroscopic signal emitted by the AmB’s chromophore group, which indicates AmB content decrease in the nail lacquer (Figure 1A,B).

In addition, it is possible to observe that the AmB concentration decay followed two different profiles: (i) a fast decay between 0 and 30 days (100.0 ± 0.0% to 53.0 ± 0.5%, respectively) and (ii) a slow decrease from 30 to 90 days (53.0 ± 0.5% to 21.0 ± 0.4%, respectively) (Figure 1B). Moreover, it is also important to notice that the first 10% of AmB decrease occurred after seven days of storage. In a preliminary product stability assessment, this might indicate that the AmB-loaded nail lacquer presents a stability profile suitable for an extemporaneous product. Therefore, because of this limited stability, the formulation may be easily produced for intended immediate use [53].

### 3.2. Physicochemical Characterization

The physicochemical characteristics of a system intended for drug delivery across the nail are fundamental to support its efficacy and safety and to predict its usability and acceptability by the patients with onychomycosis. Hence, the following physicochemical analyses were performed on the AmB-loaded nail lacquer.

Initially, the visual analysis allowed us to observe that the obtained system was (i) a homogeneous and viscous liquid, (ii) with translucent and yellowish aspect (due to the AmB characteristics) and (iii) with an absence of visible precipitates. Additionally, the physicochemical characterization results are shown in Table 3.

The obtained drying-time and non-volatile-content results were approximately 200 s and 20.00% *_w/w_*, respectively, which showed no significant difference (*p* > 0.05) over time. Furthermore, the water-resistance result was approximately 2.00% *_w/w_* of weight loss in water medium over 90 days. The in vitro adhesion results indicated that the AmB-loaded nail lacquer adhesivity presented score zero (0) in all performed analyses. Additionally, the tested sample did not show any blistering over time in the blush tests. These results allowed us to approve (pass) the sample for this parameter.

All the performed physicochemical analyses and the obtained results were based on a solid and translucent film formation. Herein, it is important to notice that in those experiments containing water, a whitish film instead of a translucent one was observed when the film was not completely dried in a previous step. Such phenomenon could be indicative of a chemical incompatibility between the nail-lacquer formulation and the water. This could be explained by the difference in polarity and dielectric constants between the lacquer solvent mixture and the water [54]. Hence, in these cases, the experiments were repeated until the achievement of a translucent film.

### 3.3. In Vitro Antifungal Activity Evaluation

In order to evaluate the in vitro antifungal activity in standard strains of *Candida* spp. (*Candida albicans*, *C. glabrata*, *C. dubliniensis*, *C. tropicalis* and *C. parapsilosis)*, the agar well diffusion assay was performed. This in vitro method is based on the diffusion of the samples and controls across the agar from a well, allowing a halo formation, which indicates antifungal effect [55]. Furthermore, these strains are the most prevalent *Candida* spp. found in onychomycosis cases. Standard *C. albicans*, *C. parapsilosis* and *C. tropicalis* were chosen due to their correlation with the clinical findings [2,13,14,15,16,17,18,19].

**Figure 2:**
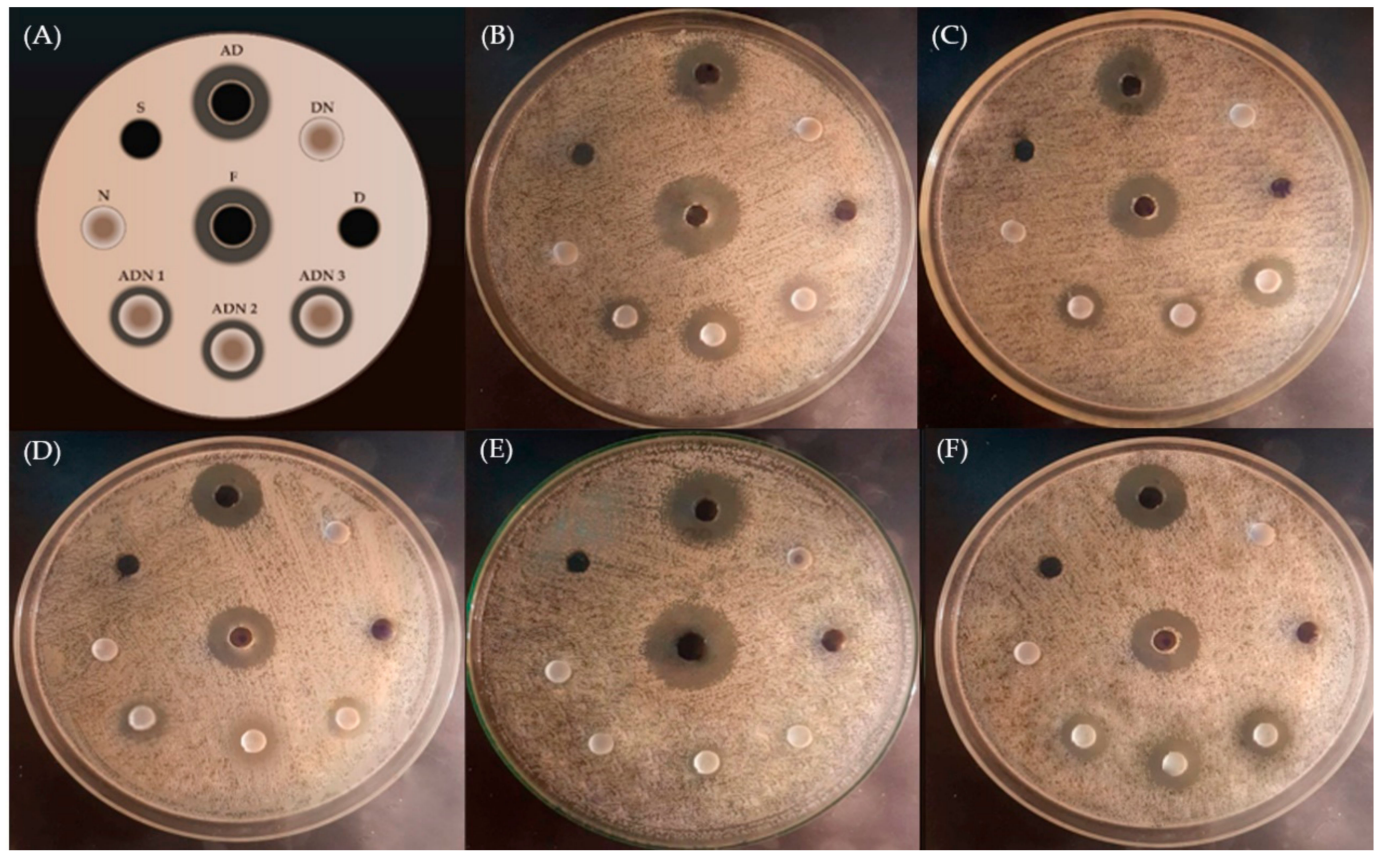
Qualitative evaluation of the in vitro antifungal activity by the agar well-diffusion method. (**A**) Samples application scheme, (**B**) *Candida albicans* ATCC 90028, (**C**) *C. glabrata* ATCC 2001, (**D**) *C. tropicalis* ATCC 13803, (**E**) *C. dubliniensis* CBS 7987 and (**F**) *C. parapsilosis* ATCC 22019. ADN, Amphotericin B–loaded nail lacquer; AD, Amphotericin B–DMSO; F, Fungizone^®^; DN, DMSO nail lacquer; D, DMSO; N, nail lacquer; S, saline solution.

Hence, the in vitro antifungal activity results showed qualitative (Figure 2) and quantitative (Table 4) data based on the inhibition halo formation surrounding the tested samples. The qualitative evaluation was performed to observe the diffusion ability of the samples and controls in the agar surface and then, to confirm if this chosen method was suitable to evaluate the in vitro antifungal activity of the AmB-loaded nail lacquer. This qualitative assessment was needed to ensure the suitability of this approach, since previously performed experiments by our research group (data not shown) indicated that, due to the nail lacquer’s physicochemical characteristics, the microdilution broth method (gold standard to evaluate the antifungal activity and minimum inhibitory concentration) was unsuitable for the nail-lacquer samples. The water-insoluble solid sample impairs its serial dilution in broth, which, along with the absence of an effective stirring, compromises the drug homogenization into the wells/medium.

Regarding Figure 2, the positive controls (samples named as F and AD) showed radial absorption in the agar medium with inhibition halo formation for all tested strains. In contrast, the negative controls (samples named as S and D) did not present any halo formation, ensuring the reliability of the test. Furthermore, no inhibition halos for N and DN appeared, suggesting an absence of antifungal activity for these controls. However, the AND showed a remarkable inhibition halo for *Candida albicans* ATCC 90028 (Figure 2B), *C. glabrata* ATCC 2001 (Figure 2C), *C. tropicalis* ATCC 13803 (Figure 2E) and *C. parapsilosis* ATCC 22019 (Figure 2F).

These results also indicate that the nail-lacquer formulation produced an opaque and whitish film, which could be attributed to the interaction between the nail-lacquer formulation and the water, presented in the agar medium. This formed interface could be where the AmB is anchored for release to the agar media and to promote its antifungal effect, leading to the formation of an inhibition halo. Furthermore, the qualitative data showed that this method was suitable to predict the AmB-loaded nail lacquer’s antifungal activity, since inhibition halos for the positive controls were found.

The quantitative evaluation was performed by measuring the inhibition zones diameters following the classification proposed by the CLSI guideline: *Epidemiological Cutoff Values for Antifungal Susceptibility Testing (M59)* [56]. This document establishes the antifungal susceptibility breakpoints for *Candida* spp. as follows: (i) susceptible = inhibition halo formation ≥ 15 mm (mm) in diameter; (ii) susceptible dose-dependent = inhibition halo formation between 14 and 10 mm in diameter; and (iii) resistant = inhibition halo formation ≤ 9 mm in diameter. Then, the obtained results were expressed as the average (n = 3) diameter in millimeters and did not include the well diameter. Additionally, the AmB concentration used in this assay (0.02 mg/mL) was determined based on the Clinical and Laboratory Standards Institute (CLSI) guideline *Method for Antifungal Disk Diffusion Susceptibility Testing of Yeasts (M44-A2)* [50]. The obtained results can be observed in Table 4.

Table 4 shows that the negative controls, D, N, DN and S, did not promote inhibition zones. As a result, any inhibition halo formation could be attributed mainly to the loaded drug. Additionally, ADN was able to produce inhibition zones for *Candida albicans* ATCC 90028, *C. glabrata* ATCC 2001, *C. parapsilosis* ATCC 22019 and *C. tropicalis* strains ATCC 13803 and did not show any inhibition zone for the *Candida dubliniensis* CBS 7987 strain (Table 4), evidencing the antifungal activity of the AmB-loaded nail lacquer. On the other hand, these results were less expressive compared to the positive controls (AD and F).

Overall, the obtained results indicated that *C. albicans* ATCC 90028 remained between susceptible and susceptible dose-dependent; *C. parapsilosis* ATCC 22019 was susceptible; *C. glabrata* ATCC 2001 and *C. tropicalis* ATCC 13803 were susceptible dose-dependent; and *C. dubliniensis* CBS 7987 was resistant to the AmB-loaded nail lacquer. Therefore, these results reinforce the hypothesis that the AmB-loaded nail lacquer could be a promising extemporaneous dosage form with a remarkable antifungal effect against different *Candida* species related to the onychomycosis.

Finally, it is known that AmB is a well-established antifungal and that it displays adequate activity against a broad variety of fungi. Although this work focuses on *Candida* spp. induced onychomycosis, this assay was also a proof of concept that the nail lacquer allows the pharmacological action of AmB. Therefore, these results indicate that the AmB-loaded nail-lacquer formulation could also be further investigated for different types of onychomycosis etiological agents.

### 3.4. AmB-Loaded Nail Lacquer’s Drug Release and Ex Vivo Permeability

**Figure 3:**
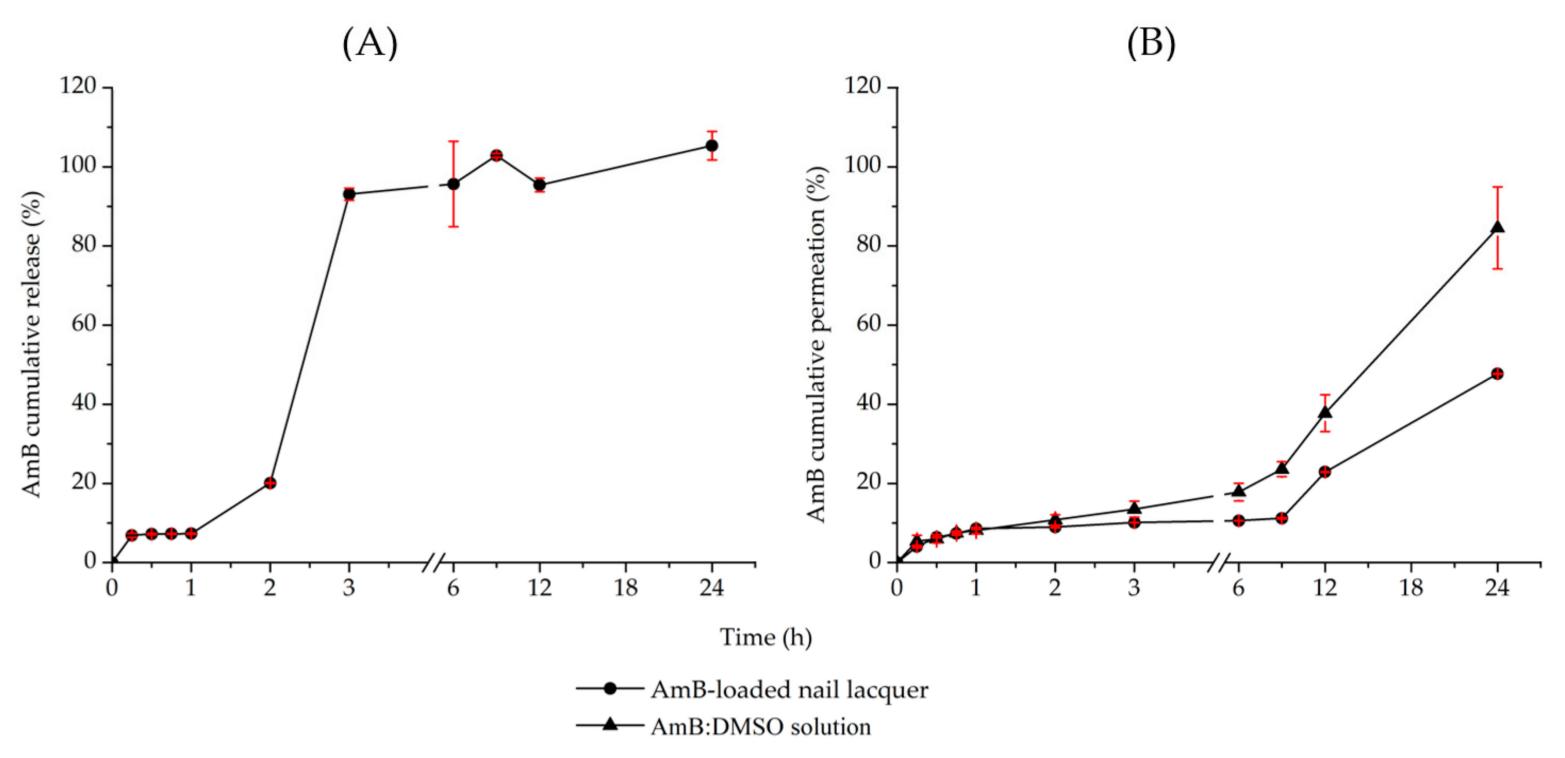
AmB in vitro release and ex vivo permeation profiles from AmB-loaded nail lacquer. (**A**) AmB’s cumulative release percentage in phosphate saline buffer medium. (**B**) AmB’s cumulative permeation percentage across an ex vivo nail model from bovine hooves.

An in vitro drug release assay was performed to evaluate the AmB release from the dry AmB-loaded nail lacquer’s polymeric film and to ensure that the findings from the in vitro antifungal activity evaluation were provided from the delivered AmB from the nail-lacquer film. Phosphate saline buffer (0.1 M pH 7.4) containing Tween^®^ 80 (2%) was used as the in vitro medium due to its ability to mimic the characteristics from human body fluids and to prospect further clinical studies. Results are displayed in Figure 3A as cumulative percentage of AmB released in the medium.

Figure 3A shows that the drug release (7.30% ± 0.15%) started from 0.25 h and maintained a similar profile up to 1 h. Subsequently, a slight drug release increase (+12.77 ± 0.5%) occurred between 1 and 2 h, followed by a burst event (+73.01 ± 1.42%) between 2 and 3 h, which represented 93.08 ± 1.52% of the AmB cumulative release. Moreover, ≅ 100% of cumulative drug release was observed after 9 h of experiment, which remained constant on a plateau near 100% up to 24 h, ensuring that the total drug content was released. Figure 3B displays the AmB permeation behavior across an ex vivo nail model matrix. AmB permeated the nail matrix and reached the receptor medium in 0.25 h when applied as AmB-loaded nail lacquer (4.03 ± 0.32%) and AmB:DMSO solution (5.43 ± 1.46%). Regardless of the prompt release from the formulation after 2 h (Figure 3A), AmB permeated across the nail matrix at a steady rate up to 9 h (Figure 3B), reaching a total of 11.18 ± 0.16% and 23.60 ± 1.89% for AmB-loaded nail lacquer and AmB control, respectively. After 12 h, the cumulative permeation began to increase at a higher rate, displaying values of 47.76 ± 0.07% and 84.54 ± 10.34% for AmB-loaded nail lacquer and AmB control, respectively. These in vitro results suggest that AmB was successfully delivered from the polymeric solid film of the nail lacquer to the receptor medium, moving across the ex vivo model nail plate and promoting its desirable antifungal effect.

## 4. Discussion

### 4.1. Method Validation

To provide a suitable product with high potential to topically inhibit fungal infections related to onychomycosis, a commercial nail-lacquer formulation was chosen based on its composition. AmB was added to this vehicle since this drug has a remarkably broad spectrum of activity. It can act against the main fungal strains responsible for the aforementioned disease. For this purpose, AmB was previously solubilized in DMSO and then loaded in the nail-lacquer formulation.

The method validation for UV–Vis Spectroscopy was conducted to assure the reliability in quantifying AmB for further experimental protocols. First, the specificity results demonstrated no interference from the formulation excipients on AmB’s absorbance at 416 nm. Therefore, the method can be considered specific for the proposed formulation and the drug. The linear regression from the calibration curve (data not shown) demonstrated adequate linearity (R^2^ = 0.9997 ± 0.0001) in the range of 0.12–1.11 µg/mL because the correlation coefficient was not statistically different from 1. The model equation (y = 0.9700x + 0.0262) was suitable to generate a linear relation between the response and the concentration.

The amount of scatter in the results was assessed by the precision studies. According to the FDA, the acceptance criteria for drug products are % RSD ≤ 3 [57]. Within-day, inter-day and inter-analyst precision results were less than 1%, which indicated the high precision of the developed method. Such values suggest that this easy-to-conduct method could be performed over different days and by different trained analysts without compromising the results. Concerning the accuracy of the method, the recovery percent represents the closeness of the quantified concentration to the true value. In this study, samples at the target concentration were quantified for the recovery determination and demonstrated to be accurate (recovery of 102.07 ± 1.23%) according to FDA non-compendial guidance (acceptable range of 95–105%) [57].

Regarding the method’s robustness, the absorbance of samples after variations in temperature and solvent manufacturer were not statistically different (*p*-value > 0.05). These results indicate that the method is reliable even in adverse conditions, such as environmental and analytical temperature variations, solvent purity and manufacturer changes. The DL means the lowest concentration safely distinguishable from the baseline noise. Accordingly, the calculated DL of 0.073 µg/mL stands below the lowest concentration point from the calibration curve (0.12 µg/mL). On the other hand, the QL is the lowest concentration quantifiable by the method with precision and accuracy. The calculated QL of 0.221 µg/mL is higher than the lowest sample concentration from the linearity curve. In this case, it is reasonable to propose a suitable range that displays not only linearity, but also precision and accuracy [58]. Hence, the analytical method range was determined to be 0.221–1.11 µg/mL, as it meets all of the literature’s acceptance criteria. Based on the overall results, the AmB quantification method was successfully validated, allowing the reliable quantification of AmB in the subsequent experimental steps.

### 4.2. AmB-Loaded Nail Lacquer’s Quantification and Stability Evaluation

The formulation components and the incorporation method resulted in an AmB loading capacity in the nail lacquer of approximately 100%. This result could be attributed to the high AmB solubility in the DMSO and, also, in the mix of organic solvents present in the colorless nail-lacquer formulation. Indeed, the DMSO is an organic solvent that presents a noteworthy AmB solubilization ability (30–40 mg of drug per DMSO mL), as described on the Analytical profiles of drug substances: Amphotericin B [59]. Additionally, this solvent was chosen due to its potential to improve the AmB permeation across the nail, once it is commonly used as a permeation enhancer in a wide concentration range [60].

Several marketable products for healthcare and drug delivery applications, mainly sustained-release formulations, use DMSO in their composition [60,61]. Regarding the improvement of drug permeation, the literature describes the DMSO ability to improve the transdermal delivery of diclofenac, ciclosporin, timolol and several other drugs [60,61]. In fact, a marketable ciclopirox-based nail lacquer (Micolamina^®^) has DMSO in its formulation as a permeation enhancer [62]. Therefore, the use of this solvent in a specific and safe range is well supported in the pharmaceutical development for these products.

Furthermore, the previous AmB solubilization in DMSO and loading in a nail-lacquer organic media stands out as a simple and feasible incorporation technique. Other authors also used this approach to incorporate drugs in nail lacquers, as Josh et al. (2015) [48] and Akhtar et al. (2016) [63]. They used *n*-butanol and isopropyl alcohol as solvents for isotretinoin and tolnaftate, respectively, ensuring that this technique is suitable for the produced formulation.

After the AmB loading in the nail-lacquer formulation, it was imperative to ensure the drug stability in the organic media, which was assessed by the AmB quantification over 90 days. The results revealed a significant decrease in the AmB UV–Vis spectra signal over the days, suggesting drug degradation by its chromophore modification (Figure 1A).

Moreover, a 10% decrease in the AmB content occurred after 7 days of storage at 5 ± 3 °C, whereas at the end of the 30 days only 50% of the loaded drug remained in the formulation (Figure 1B). In fact, the AmB loss appears to be a bilinear process, which could be suggestive of a complex degradation process in the solvent matrix of the nail lacquer [12]. AmB degradation could be attributed to different factors. Studies from our research group have demonstrated that this molecule is susceptible to complex degradation processes in organic media according to environmental conditions and co-solutes, such as excipients [58]. Alencar et al. (2021) determined that under dark conditions, AmB undergoes autoxidation and further phenomena in a bilinear kinetics, while under light exposure, photo-oxidation accounts for most AmB loss in a pseudo-first order kinetics [58].

Overall, non-aqueous media are often responsible for triggering multi-pathway AmB degradation according to its aggregation state, which may appear as a monomer or different aggregates. This has been already reported by Lamy-Freund et al. (1993), who showed that the AmB aggregation state can influence the degradation rates and mechanisms [64].

Notwithstanding its degradation pathway, the remaining AmB concentration at day 7 (≅ 90%) could be considered proper if the AmB-loaded nail lacquer would be available as a ready-to-use extemporaneous product. Overall, the degradation profile along of the 90 days suggests that the AmB stability in the chosen nail-lacquer formulation can be further improved to lower this drug’s degradation rate in this media. However, it is fundamental to point out that this stability profile is compatible with medicines for immediate use, also known as extemporaneous products [53]. They are advantageous drug dosage forms once they can be indicated for individualized care and adapted to the patient’s specific needs. Despite showing short-term stability, they are suitable for therapeutic purposes if the product is maintained only for 7 days. In this context, we hypothesize that the product could be designed to be available as a ready-to-use polymer-based AmB solution (AmB-loaded nail lacquer). Accordingly, this formulation could be dispensed to the patient immediately after its production, in a pharmaceutical environment in a context of clinical assays or even as a marketed product in which the diluents and the AmB powder would be present.

Despite the relevance of chemical stability of AmB in the nail-lacquer formulation, it is fundamental to notice that, according to the Clinical and Laboratory Standards Institute (CLSI) at the guideline *Epidemiological cutoff values for* in vitro *susceptibility testing of Candida* spp. *(M59)* [56], no more than 2 μg/mL of AmB content is needed for an effective antifungal activity against *Candida* spp. (*C. albicans*, *C. glabrata*, *C. parapsilosis*, *C. tropicalis* and others). Based on this rationale, regardless of the high degradation rate of AmB in the nail-lacquer formulation, after 7 days of storage, the drug content remains 9-fold higher than the minimum recommended (≅18 μg/mL of AmB) and, then, suitable to promote its fungicidal activity.

Hence, it is possible to infer that the AmB-loaded nail lacquer was successfully produced, and, previous to its antifungal activity investigation, its physicochemical properties need to be evaluated.

### 4.3. Physicochemical Characterization

The physicochemical characteristics evaluation is an important step to ensure the quality of formulations intended for therapeutic purposes. Although nail lacquers are not commonly used for this goal, this system was chosen due to its ability to overcome the main drawbacks of oral treatment, which are side effects associated with the use of multiple oral medicines and the need to maintain the antifungal dosage for a long treatment time [10,23]. Although its use appears as a new approach to overcome the limitations of the oral treatment, the use of nail lacquers could also display certain drawbacks. It is possible to mention the low permeability of the marketed formulations, as demonstrated by Chiacchio et al. (2013) in their study about a nail lacquer containing amorolfine 50 mg/mL and ciclopirox 80 mg/g, which required constant reapplications or even the nail abrasion process to facilitate the drug permeation into the infectious site [24]. Hence, the produced formulation was designed to enable the use of AmB in the onychomycosis management due to its immediate and higher fungicidal activity than the aforementioned molecules. This approach may reduce the need for constant reapplications and long treatment times and, as a result, improves patient compliance [28,29,30].

The AmB-loaded nail lacquer showed a yellowish appearance attributed to the AmB presence. Not to mention, a translucent and uniform aspect was observed. Further, the formulation did not exhibit blistering or peeling off along the physicochemical evaluation study. It is important to note that the visual aspect is an important parameter due to the cosmetic appeal of this formulation. Moreover, it can improve patient compliance [48]. Moreover, to assess the nail lacquer usability and to predict the aspect during usage, the drying-time analysis was performed.

The drying time is defined as the time required to a dried solid film be formed [48,65]. If the drying time of the nail lacquer is short as a result of high drying ability, it may lead to a fast film formation on the nail surface during the application of the nail lacquer (indicative of a poor flow), leading to an inhomogeneous and irregular film formation [48,63,65]. On the other hand, if the drying time is too high, it indicates high fluidity, which leads to difficulty in application, once the product may spread to the nails edges and be lost [48,63,65].

Therefore, the drying time for nail lacquers has been reported to be from 30 to 90 s [48,63,65]. On the other hand, Pittrof et al. (1992) [66] and Tabara et al. (2015) [25] reported that the marketable formulations loaded with amorolfine showed suitable drying times between 180 and 300 s. In this work, the AmB-loaded nail lacquer showed a drying time of approximately 200 s, which may suggest that it can be acceptable and suitable for application on nails.

Moreover, this result can be associated with the nail-lacquer composition (Table 1). Several literature reports, as the studies of Josh et al. (2015) [48], Khattab and Shalaby (2018) [65] and Akhtar et al. (2016) [63] evaluated the effect of different solvents (*n*-Butanol and isopropyl alcohol), film-forming agents (Eudragit^®^ RS 100 and Eudragit^®^ RLPO), plasticizers (polyethylene glycol, glycerol and triacetin) and others excipients (thioglycolic acid and Tween^®^ 80) in their nail-lacquer-formulation drying time. These authors attributed the drying time results to the boiling points and different vapor pressures of the used excipients and their concentration in the formulations. Therefore, according to the cited studies, it can be suggested that the obtained drying time for the AmB-loaded nail lacquer presented in this work can be attributed to the presence of butyl acetate, toluene, ethyl acetate, dimethyl sulfoxide and alcohol used in the formulation, since they can modulate this parameter.

Furthermore, the non-volatile content in the AmB-loaded nail lacquer was also evaluated. This test aims to analyze the total amount of solid compounds that remain in the dried film after the solvent evaporation, providing data regarding the quality of the formed film [48,63,65]. In this concern, the literature reports suggest that the obtained non-volatile compounds should provide a dry and cohesive film to completely cover the nail surface [48,63,65]. Hence, the ideal non-volatile content should be at least 20.00% *_w/w_* [48,63,65].

Accordingly, Josh, et al. (2015) demonstrated that the non-volatile contents were causally related to the film-forming agent concentration. Indeed, they observed an increase in this parameter according to the forming agent concentration [48]. Similar results were reported by Khattab and Shalaby et al. (2018), who obtained 57.32 ± 0.31% of non-volatile compounds for the formulation containing the highest concentration of Eudragit^®^ (E-RLPO) [65]. Hence, the non-volatile content obtained by the AmB-loaded nail lacquer (approximately 20.00% _*w/w*_) herein produced shows to be in agreement with the literature reports, suggesting a suitable film formation that may be able to completely cover the nail surface.

Moreover, the use of nitrocellulose (film forming agent), tosylamide formaldehyde (resin) and dibutyl phthalate (plasticizing agent) may contribute to this result. It is important to point out that the use of a marketable nail-lacquer formulation to load the AmB solution could also be contributing to the finding of the ideal non-volatile content herein presented. Therefore, the data regarding the drying time test and the non-volatile content allow us to suggest that the produced formulation presents suitable properties concerning the solid film formation [48,63,65].

Furthermore, the AmB-loaded nail lacquer’s water-resistance and blush tests were also conducted since these parameters play an important role in product usability. Indeed, after administration and drying, the solid film can remain in the nail surface for a time period. The exposure to environmental factors can affect its integrity or even lead to the film erosion. Among those factors, the water can be highlighted, since the formed film may uptake water molecules and increase the surface erosion, leading to a weight loss or even to the solubility of the film and/or the drug. Therefore, the water-resistance test stands out as a fundamental tool to evaluate the quality of the formed solid film [48,63,65].

Accordingly, the water-resistance result (near to 2.00% *_w/w_* of weight loss) indicates that the formed solid film was not affected by water after 24 h of contact. Similar results were reported by Josh et al. (2015) [48], Akhtar et al. (2016) [63], and Khattab and Shalaby (2018) [65], who developed therapeutic nail lacquers with the aim of delivering isotretinoin, tolnaftate and ciclopirox, respectively. Murdan et al. (2015) performed a study emphasizing the importance of the water-resistance from therapeutic nail lacquers on drug retention and permeation, which are important parameters for drug availability at the application site [49].

In fact, water-soluble components in nail-lacquer formulations may play an important role both in the water-resistance and in the blush test, as demonstrated by Khattab and Shalaby (2018). These authors reported a decrease in water-resistance when triacetin (a water soluble plasticizer) concentration was increased [65], which lead to the polymer matrix erosion and, as a result, drug loss. Accordingly, it is possible to suggest that the obtained results were influenced by the nail lacquer’s composition (Table 1) once water-insoluble components prevent the film weight loss and improve the drug residence in the targeted site [65].

Notwithstanding the similarity to the previous assay, the blush test was performed to assess the nail-lacquer film’s physical changes, such as blistering or peeling off, after the water exposition [48,63]. Therefore, the obtained results suggest the AmB-loaded nail lacquer’s usability for long periods, since a noteworthy water-resistance and no blistering or peeling off were recorded.

Finally, the in vitro adhesion test was also assessed in order to provide data regarding the AmB-loaded nail lacquer’s adherence at the nail’s surface [49]. All tested samples showed suitable in vitro adhesion (100%). This property is fundamental to achieve the management of onychomycosis by therapeutic nail lacquers. In fact, adhesion is necessary to assure that the solid film remains adhered to the nail surface until the loaded drug can be released for retention/permeation and reach therapeutic concentrations in the infection site [48,67].

In addition, the AmB loading in this formulation was not able to promote changes in the physicochemical properties of the blank nail lacquer (a commercial product), once all the performed tests provided similar results from the blank nail lacquer’s physicochemical characterizations previously performed (data not shown). Therefore, the overall results suggest that the produced AmB-loaded nail lacquer’s formulation, stored at 5 ± 3 °C, presented suitable physicochemical properties and usability that remained unchanged over 90 days. Further, its therapeutic activity was assessed by the in vitro antifungal activity against different strains of *Candida* spp., related to onychomycosis.

### 4.4. In Vitro Antifungal Activity Evaluation

The evaluation of the in vitro antifungal activity is an important approach to determine the fungal strains’ susceptibility against the produced AmB-loaded nail lacquer. In this regard, several methodologies can be used to this investigation, among which the microdilution broth method and agar diffusion method can be highlighted. Then, as the broth microdilution assay was unsuitable for the AmB-loaded nail lacquer, as previously described, the agar diffusion method was chosen [55,68].

The results revealed that the used positive controls, AmB/DMSO and Fungizone^®^, were able to inhibit the fungal growth. Indeed, AmB marketable formulations, such as Fungizone^®^, are widely used as positive controls in such assays due to their proven effectiveness in the pharmacological field. Furthermore, these formulations can provide data, even by in vitro tests, that can be correlated to the biological conditions [69].

Additionally, the antifungal activity for Fungizone^®^ and AmB/DMSO solution were, at least, 2 to 2.5-fold higher than the one found for the AmB-loaded nail lacquer (Figure 2 and Table 4). These data can be attributed to the physical state of the Fungizone^®^ and AmB/DMSO, which are a micellar dispersion and solution, respectively. Due to its liquid state, the AmB diffusion across the solid agar medium is facilitated, in contrast to the viscous and polymeric matrix of the nail-lacquer formulation. However, as previously discussed, both dosage forms are not suitable for treatment of onychomycoses due to their low retention time at the nails.

On the other hand, the AmB-loaded nail lacquer displayed a remarkable antifungal activity against the main *Candida* spp. strains responsible for the onychomycosis in at-risk patients. Ordinarily, patients who are infected by the human immunodeficiency virus (HIV), patients with diabetes, with acquired immunodeficiency syndrome (AIDS) and with other types of immunosuppression [5,13].

These data can be associated with the suitable characteristics of the nail-lacquer formulation and, mainly, with the use of the AmB as an antifungal drug. This molecule’s excellent fungicidal activity against the onychomycosis-causing strains was already demonstrated by Lurati et al. (2011) [31,70]. These authors showed, by in vivo assays, that AmB is an effective drug to treat onychomycosis and suggested that the use of DMSO as a permeation agent can improve this activity since it allows the availability of the free drug [31,70].

In addition, it is important to note that some literature reports described that several standard *Candida* species, mainly clinical isolated and/or mutated strains, can be viable even after 48 h of contact with 2 µg/mL of an AmB solution in the killing curve assay [42,43]. These data reinforce the hypothesis that it is possible to observe, by in vitro experimental assessments, that some *Candida* spp. strains may not be killed by AmB. Therefore, the resistance found for *C. dubliniensis* was not a weakness of the system. This phenomenon can be related to virulence factors and fungal genomic characteristics. In conclusion, these findings reinforce the remarkable antifungal activity of the produced AmB-loaded nail lacquer.

The obtained results are also in agreement with the study performed by Magaldi et al. (2004) [55], who evaluated, by the agar well diffusion method, the response of 180 *Candida* spp. clinical isolates, including *C. albicans*, *C. glabrata*, *C. tropicalis*, *C. dubliniensis* and *C. parapsilosis*, against the AmB. In this study, the drug was diluted in polyethylene glycol (PEG-400) at 1.25 mg/mL and 25 μg of AmB per well was applied to evaluate the strains’ susceptibility. From the 180 strains isolates, only two *C. albicans* were considered resistant and 1 *C. glabrata* was considered susceptible dose-dependent, showing the broad antifungal spectra of AmB [55]. Additionally, our findings are also supported by the study conducted by Arendrug, et al. (2001), who tested 119 isolates of *Candida* spp. (*C. albicans, C. tropicalis, C. glabrata* and *C. parapsilosis*) from which 117 strains were susceptible to AmB and only two isolates (1 *C. glabrata* and 1 *C. parapsilosis*) were considered as susceptible dose-dependent [68]. Therefore, the obtained results and the literature reports reinforce that the use of AmB in a nail-lacquer formulation could be an excellent approach to the management of *Candida* spp. induced onychomycosis.

Moreover, from an etiologic point of view, Lipner and Scher (2019) suggested that the *Candida* spp. are responsible for 10–20% of the onychomycosis occurrence worldwide [71]. However, it is known that *Candida* spp. prevalence in different Countries is variable and can increase according to the population’s age, work habits, health and other factors. In fact, Andrés and Alexandro (2020) determined from a series of worldwide literature reports that onychomycosis caused by *Candida* spp. display high prevalence, usually greater than 50% [13]. Furthermore, they highlight that onychomycosis induced by *Candida* spp. in severely immunosuppressed patients is more prevalent than previously thought by the medical community [5,13].

Based on this, the observed antifungal activity of the AmB-loaded nail lacquer is a promising result because the produced formulation was able to act against strains with a remarkable presence on the onychomycosis etiology. Not to mention, in a retrospective study over 17 years, Geizhals, et al. (2020) evaluated the diagnostic testing and medical management for onychomycosis including 1774 patients, among which 1175 received oral treatment (66.2%) and 599 received topical treatment (33.8%) [72]. The study showed that the terbinafine tablet/capsule (93.4%) and ciclopirox nail lacquer (51.3%) were the most commonly prescribed products for oral and topical treatment, respectively. Hence, it was possible to suggest that the produced AmB-loaded nail lacquer could be a promising product to improve the current arsenal intended for the onychomycosis treatment.

Finally, it is important to highlight that the polymeric nail-lacquer matrix may modulate the AmB release at different rates according to the affinity between the drug and the film, the type of the crosslinking formed and/or the medium ability to extract the drug loaded into the film. These factors can also be associated with the observed *Candida dubliniensis* CBS 7987 resistance. In addition, this behavior could be related to the intrinsic characteristics of this strain, such as the growth speed, once the positive controls showed inhibition halo formation. Therefore, it is possible to hypothesize that the *Candida dubliniensis* CBS 7987 would not be resistant if the formulation releases 100% of the AmB since the beginning of the experiment. Then, drug release and drug permeation experiments were conducted and are shown in the following section.

### 4.5. AmB-Loaded Nail Lacquer’s Drug Release and Ex Vivo Permeability

The drug release assay is an important tool to ensure the therapeutic usefulness of the developed/produced dosage forms. Indeed, every drug must be released from the formulation to reach, at desirable concentrations, the infected site and promote its pharmacological effect. As discussed in the previous sections, the AmB-loaded nail lacquer presented suitable physicochemical properties and a remarkable antifungal activity against several *Candida* spp. strains related to onychomycosis. However, it displayed limited activity against *C. dubliniensis* CBS 7987. This fact can be attributed, among several factors, to the drug release from the nail-lacquer formulation. Accordingly, to ensure the suitable AmB release from the used formulation, and to investigate the observed resistance from *C. dubliniensis* CBS 7987, the AmB-loaded nail lacquer’s drug release assay was performed.

Figure 3A shows that more than 90% of the AmB content was released after 3 h of assay. This fact can be associated to the AmB solubilization prior the loading into the nail lacquer, which ensured the drug molecular dispersion in the formulation. In addition, it was possible to confirm that complete AmB release did not happen at time 0, which could help explain the hypothesis for the *C. dubliniensis* CBS 7987 reduced susceptibility. Furthermore, it is important to note that, since AmB was released in a burst-like behavior around 3 h, it is possible that this drug is not present in a complex arrangement or organized structure and, therefore, was easily released from the nail-lacquer formulation.

Accordingly, Kim et al. (2010) [51] showed a similar result from a polymeric inclusion complex of AmB delivered by a gel, from which the AmB release plateau was achieved at near 6 h. However, differently from our findings (100% of AmB release), the AmB release obtained by Kim et al. (2010) was not 100% (pH 7.4), which can be associated to the different obtained structures. Therefore, it might be inferred that after the nail lacquer drying, the AmB remains free and homogeneously distributed in the matrix, making it available to be released.

Although release data suggested that AmB has the potential to be released from the nail lacquer to reach the site of action, it was still unclear if the drug could overcome the target biological barrier, i.e., the nail matrix, and reach the infection site. Hence, a permeability study was performed by using bovine hooves as an ex vivo animal model. Such model was chosen because the hooves are formed by layers of intensely adhered keratinized cells rich in disulfide bridges (-S-S-), as are the human nails.

Figure 3B shows that in 24 h AmB permeated the nail matrix from the solution in DMSO (84.54 ± 10.34) and from the AmB-loaded nail lacquer (47.76 ± 0.07%). The high permeability from the AmB in solution can be attributed to the DMSO mechanism to displace water from the biological tissues. Further, it disrupts lipidic and protein structures, as described by Rowe, Raymond and Sheskey (2009), who mentioned that DMSO concentrations lower than 15% can increase the drug permeation in rich keratin protein matrixes, such as the nails [60].

The AmB permeability of 47.76 ± 0.07% represents only half the AmB dose loaded in the nail lacquer. However, the amount that permeated the nail matrix represents a 5-fold higher concentration than the minimum recommended to promote a suitable antifungal activity. This result is supported by the performed in vitro antifungal assay, in which AmB samples (2 μg of drug/100 μL of medium) were tested, and four from five *Candida* spp. were susceptible dose-dependent.

Therefore, these lower permeability results can be attributed to the complex media presented on the nail-lacquer formulation (polymeric organic solution) and how AmB behaves in relation to its physical and ionic state. AmB appears as a monomer or different aggregates according to the media, concentration and temperature. This drug contains a carboxyl group (pKa 5.5) and a primary amine group (pKa 10). Hence, the ionized groups result in a zwitterion [58]. In this context, Baswan et al. (2016) demonstrated that the effective diffusivities of cations in the nail were three times greater than those of anions of comparable sizes, highlighting the strong charge selectivity of the nail plate for permeation and ionic diffusion. This study also revealed that ionized molecules with radii above 5 Å (≥ 340 Da) will require excipients that promote permeation or mechanical alteration of the nail plate to overcome this barrier to reach the nail bed [26]. Accordingly, the organic-based medium of the nail lacquer and the excipients could modulate AmB ionization and aggregation, which implies that further formulation improvements may be necessary if higher permeability is needed in prospective in vivo studies.

## 5. Conclusions

The management of onychomycosis induced by *Candida* spp. is still challenging due to the complexity of the disease etiology, which requires a suitable formulation and a potent antifungal drug. In this study, the produced AmB-loaded nail lacquer showed itself as a promising product to contribute to this issue once it was able to load 0.02 mg/g of AmB and presented a stability profile similar to extemporaneous dosage forms. Moreover, the AmB-loaded nail lacquer presented suitable physicochemical characteristics, such as desirable water-resistance, drying time, adhesion and no physical deformity, which infers its usability. The antifungal activity showed noteworthy inhibition of several standard *Candida* spp. These results encourage further studies of formulations intended for the management of the growing public health concern that is the onychomycosis induced by *Candida* spp. Finally, the nail lacquer was able to release around 90% of its AmB content in 3 h and a permeation study that used an ex vivo model of nail matrix showed that 47.76 ± 0.07% of the AmB permeated the nail matrix in 24 h (AmB 0.010 mg/g of nail lacquer). This represents a suitable AmB amount to provide a local antifungal effect. Overall, the obtained results indicate that the AmB-loaded nail-lacquer formulation was successfully produced and showed suitable physicochemical characteristics and antifungal activity, which may contribute to its further biological investigations. Henceforward, the improvement of the AmB-loaded nail lacquer’s relevance includes directing its potential to onychomycosis caused by different fungi, such as dermatophytes, especially *Trichophyton rubrum*.

## Figures and Tables

**Table 1:** Description of the AmB-loaded nail lacquer composition.

Formulation	Component	Function	Solubility *	Log P *	Molecular Weight (g/mol) *
Colorless nail lacquer	Nitrocellulose	Film-forming agent	Immiscible with water [32]	3.1 [32]	999.4 [32]
	Tosylamide Formaldehyde Resin	Resin	N/A	N/A	201.25 [33]
	Dibutyl Phthalate	Plasticizing agent	Soluble in most organic solvents, such as carbon tetrachloride, ethanol, ethyl ether, benzene, acetone and oils [34]	4.50–4.72 [34]	278.34 [34]
	Butyl Acetate	Solvent	Soluble in ethanol, ethyl ether, acetone, chloroform, propylene glycol and in most hydrocarbons [35]	1.78–1.82 [35]	116.16 [35]
	Toluene	Solvent	Soluble in alcohol, chloroform, ether, acetone, glacial acetic acid, carbon disulfide, benzene, diethyl ether [36]	2.69–2.73 [36]	92.14 [36]
	Ethyl Acetate	Solvent	Soluble in water, ethanol, ethyl ether; acetone, benzene, chloroform, glycerol, fixed and volatile oils, oxygenated and chlorinated solvents [37]	0.73 [37]	88.11 [37]
	Alcohol	Solvent	Soluble in chloroform, ether, glycerin and water [38]	(−0.31)–(−0.32) [38]	46.07 [38]
	Tocopherol	Antioxidant agent	Practically insoluble in water, freely soluble in acetone, ethanol, ether and vegetable oils [39]	10.7–12.8 [39]	430.7 [39]
AmB/DMSO solution	Dimethyl sulfoxide	Solvent for the antifungal drug	Soluble in water, ethanol, acetone, ether, carbon tetrachloride, ethyl acetate, chloroform and benzene [40]	(−0.6)–(−1.35) [40]	78.14 [40]
	Amphotericin B	Antifungal drug	Insoluble in water at pH 6–7; solubility in water at pH 2 or pH 11 about 0.1 mg/mL; water solubility increased by sodium desoxycholate. In N,N-dimethylformamide 2–4 mg/mL, in N,N-dimethylformamide + hydrochloric acid 60–80 mg/mL, in dimethyl sulfoxide 30–40 mg/mL [41,42]	(−2.3)–0.8 [41,42]	923.488–924.10 [41,42]

N/A: not available. * Data obtained from online databases.

**Table 2:** Validation parameters.

Parameter	Result
Specificity	no interference detectable (*p*-value > 0.05)
Calibration Curve	y = 0.9701x + 0.0262
Linearity (µg/mL)	0.12–1.11
Coefficient of Determination (R^2^)	0.9997 ± 0.0001
DL (µg/mL)	0.073
QL (µg/mL)	0.221
Range (µg/mL)	0.221–1.11
Accuracy (% Recovery)	102.07 ± 1.23
Robustness	no interference detectable (*p*-value > 0.05)
Precision intra-day (repeatability% RSD)	0.69
Precision inter-day (intermediate precision % RSD)	0.69
Precision inter-analyst (intermediate precision % RSD)	0.61

**Table 3:** Physicochemical characterization of the AmB-loaded nail lacquer.

TIME	DT	N-VC	WR	IVA	BT
(days)	(s)	(% *_w/w_*)	(% *_w/w_*)	Score	Pass or not Pass
0	187 ± 04	19.64 ± 0.21	2.04 ± 0.90	Zero	Pass
7	209 ± 11	20.38 ± 1.01	2.05 ± 0.01	Zero	Pass
15	237 ± 20	18.86 ± 1.07	2.08 ± 0.19	Zero	Pass
60	220 ± 40	20.61 ± 0.57	1.59 ± 0.84	Zero	Pass
90	206 ± 14	19.85 ± 0.57	2.02 ± 0.81	Zero	Pass

Legend: AmB = Amphotericin B; DT = drying time; N-VC = non-volatile content; WR = water-resistance; IVA = in vitro adhesivity; BT = blush test; s = seconds; % *_w/w_* = weight in weight percentage.

**Table 4:** Quantitative evaluation of the in vitro antifungal activity by the agar-well-diffusion method.

Strain	Inhibition Zones (mm)						
	ADN	AD	F	DN	D	N	S
*Candida albicans* ATCC 90028	11.00 ± 3.24	26.33 ± 2.31	26.00 ± 1.73	-	-	-	-
*Candida glabrata* ATCC 2001	12.50 ± 1.31	29.33 ± 2.08	28.67 ± 1.53	-	-	-	-
*Candida tropicalis* ATCC 13803	11.83 ± 0.41	24.33 ± 0.58	24.67 ± 0.58	-	-	-	-
*Candida dubliniensis* CBS 7987	-	27.00 ± 0.00	27.67 ± 2.08	-	-	-	-
*Candida parapsilosis* ATCC 22019	13.57 ± 2.51	23.67 ± 3.21	27.67 ± 2.08	-	-	-	-

ADN, Amphotericin B–loaded nail lacquer; AD, Amphotericin B–DMSO; F, Fungizone^®^; DN, DMSO nail lacquer; D, DMSO; N, nail lacquer; S, saline solution.

## Data Availability

Not applicable.
